# Histopathological whole slide image dataset for classification of treatment effectiveness to ovarian cancer

**DOI:** 10.1038/s41597-022-01127-6

**Published:** 2022-01-27

**Authors:** Ching-Wei Wang, Cheng-Chang Chang, Muhammad Adil Khalil, Yi-Jia Lin, Yi-An Liou, Po-Chao Hsu, Yu-Ching Lee, Chih-Hung Wang, Tai-Kuang Chao

**Affiliations:** 1 Graduate Institute of Biomedical Engineering, National Taiwan University of Science and Technology, Taipei, Taiwan; 2 Graduate Institute of Applied Science and Technology, National Taiwan University of Science and Technology, Taipei, Taiwan; 3 Department of Gynecology and Obstetrics, Tri-Service General Hospital, Taipei, Taiwan; 4 Graduate Institute of Medical Sciences, National Defense Medical Center, Taipei, Taiwan; 5 Department of Pathology, Tri-Service General Hospital, Taipei, Taiwan; 6 Institute of Pathology and Parasitology, National Defense Medical Center, Taipei, Taiwan; 7 Department of Otolaryngology-Head and Neck Surgery, Tri-Service General Hospital, National Defense Medical Center, Taipei, Taiwan

**Keywords:** Cancer, Cancer screening

## Abstract

Ovarian cancer is the leading cause of gynecologic cancer death among women. Regardless of the development made in the past two decades in the surgery and chemotherapy of ovarian cancer, most of the advanced-stage patients are with recurrent cancer and die. The conventional treatment for ovarian cancer is to remove cancerous tissues using surgery followed by chemotherapy, however, patients with such treatment remain at great risk for tumor recurrence and progressive resistance. Nowadays, new treatment with molecular-targeted agents have become accessible. Bevacizumab as a monotherapy in combination with chemotherapy has been recently approved by FDA for the treatment of epithelial ovarian cancer (EOC). Prediction of therapeutic effects and individualization of therapeutic strategies are critical, but to the authors’ best knowledge, there are no effective biomarkers that can be used to predict patient response to bevacizumab treatment for EOC and peritoneal serous papillary carcinoma (PSPC). This dataset helps researchers to explore and develop methods to predict the therapeutic effect of patients with EOC and PSPC to bevacizumab.

## Abstract (summary)

**Table:** 

Measurement(s)	Therapeutic Effect
Technology Type(s)	Artificial Intelligence
Factor Type(s)	whole slide image
Sample Characteristic - Environment	pathologic primary tumor stage for ovary according to AJCC 7th edition

## Background & Summary

Ovarian cancer is the fifth most common cause of cancer death worldwide among women, accounting for more deaths than any other gynecologic cancer. According to the report by American cancer society, approximately 21,410 women will be diagnosed and about 13,770 women will die from ovarian cancer in the United State of America in the year 2021^1^. At the time of diagnosis, most of the women have advanced stage disease^2^. Despite the surgery and chemotherapy of ovarian cancer, more than 70% of the patients suffer from progressive resistance and tumor recurrence to treatment. Epithelial ovarian cancer (EOC) respresents more than 95% of ovarian cancer^3,4^. EOC is further divided into five distinct histopathological subtypes, including low-grade serous, high-grade serous, mucinos, clear cell, and endometrioid ovarian cancer. High-grade serous ovarian cancer (HGSOC) is the most common subtype of EOC, representing for over 70% of the EOCs^4,5^. Peritoneal serous papillary carcinoma (PSPC), though managed according to EOC therapeutic principles, has been variably considered as EOC counterpart^6^. Different histological types of EOC have distinct cellular origin, diverse mutational spectrum, and thus, different prognosis^7^. Even within one histological type, distinct molecular subtypes with different prognoses can be found^8^. To address these issues there is a need to better characterize these differences and find reliable biomarkers to predict therapeutic effect.

Despite many years of research, there is still lack of reliable diagnostic methods that can be detected early and suitable for screening^9^. Therefore, most women are still diagnosed in advanced stage, challenging the efficacy of therapeutic effect^10^. Nowadays, the only chance to cure the ovarian cancer is at primary treatment, and its effectiveness relies upon the disease stage and histology. 90% of the women are curable if the disease is detected and treated at an early stage, even in those with more aggressive histologic subtypes. But at the time of diagnosis, most of the women have advanced stage disease, challenging the effectiveness of debulking surgery, chemotherapy, and biologic therapy^10^. Over the last two decades, the conventional treatment for the EOC is optimal cytoreductive surgery and platnium based chemotherapy. Although approximately 80% of patients respond to first-line chemotherapy, the recurrence rate and chemo-resistance is still high^11,12^. Thus, current studies are aimed on finding new therapeutic targets. Different targeted therapy and biological drugs have been gradually introduced into clinical trials for the treatment of recurrent disease as single agents, followed by combination treatments, bring the promise of turning ovarian cancer into a controllable chronic disease^5,12^.

**Fig. 1:**
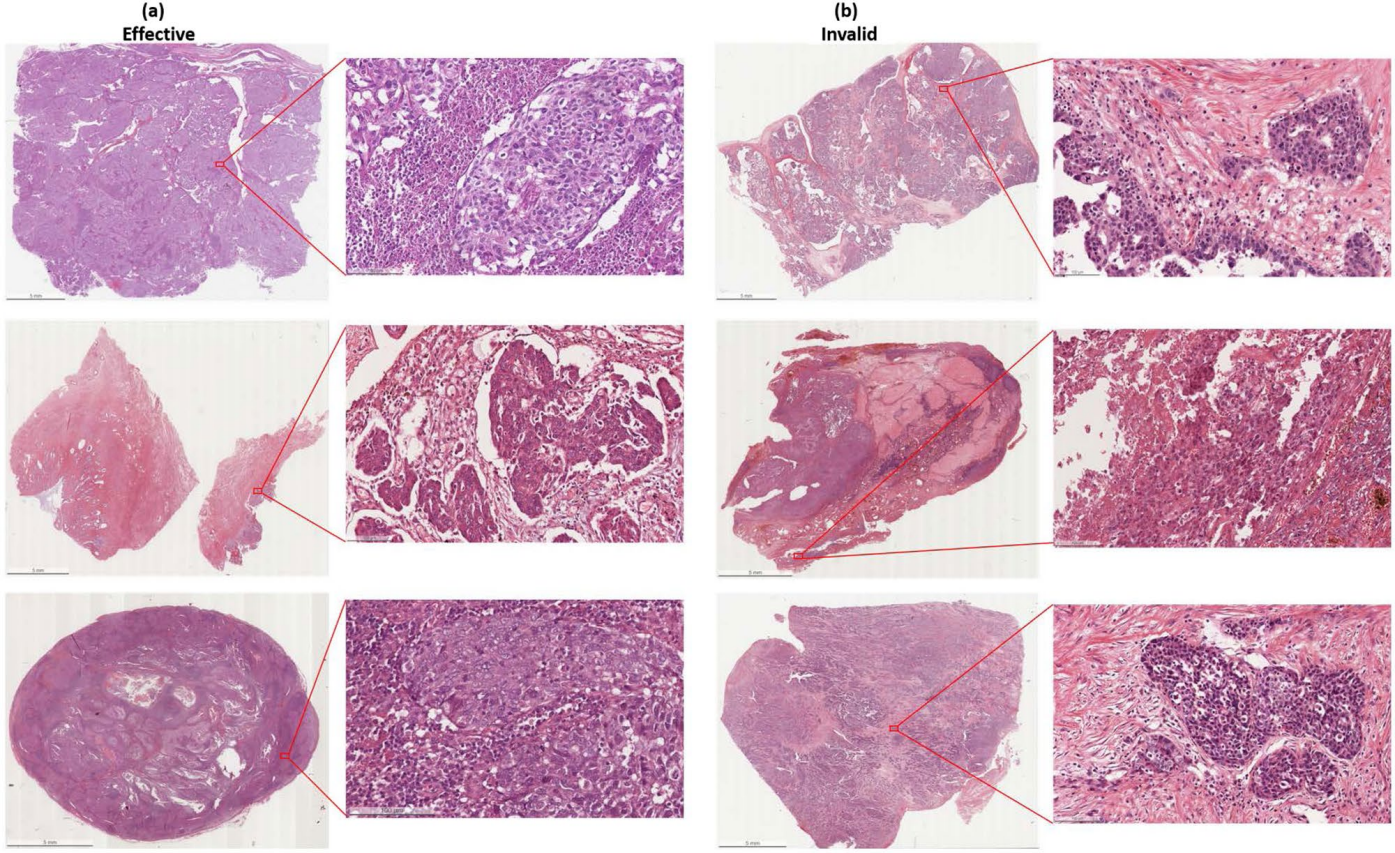
Samples of our data. (**a**) Effective bevacizumab treatment. (**b**) Invalid bevacizumab treatment.

For the treatment of recurrent disease, targeted agents have been introduced in the clinical trials to evaluate the activity as single agents, followed by the combination treatment. Over the past few years, from the introduction of concurrent bevacizumab, there has been a huge advancement in non-overlapping toxicity and improving activity^5^. Bevacizumab has been recently approved by FDA as a monotherapy for advanced EOC in combination with chemotherapy. Bevacizumab is a recombinant humanized monoclonal antibody that binds with vascular endothelial growth factor (VEGF) and neutralizes the biological activity of VEGF, and inhibits tumor angiogenesis. In 2011, according to the GOG0218 and ICON7 trial data, bevacizumab has been approved by the European Commission for first-line treatment together with standard chemotherapy in women with advanced EOC^12^. Bevacizumab has been shown to improve progression free survival for 2–4 months and in some settings also overall survival^12^. Although it is important to optimize the anti-angiogenic therapeutic effect, but anti-angiogenic agents can be very expensive and can cause serious side effects, such as delayed wound healing, hypertension, and intestinal perforation or fistula formation^5^. Therefore, considering the cost, potential toxicity, and finding that only a portion of patients will benefit from these drugs, the identification of new predictive method for the treatment of EOC remains an urgent unmet medical need. Currently, the standard diagnosis of EOC is done from the microscopic analysis of tissues section from debulking surgery, that are mounted on hematoxylin and eosin (H&E) stained glass slides^13^. Digital whole slide images (WSIs) are used to study an entire histology slide. Further, WSIs helps the pathologists to refine their decisions by performing computer-aided diagnostic (CAD) analysis. CAD analysis can automatically produce diagnostic cues that can increase the diagnostic accuracy^14–16^, while also saving time. To the authors’ best knowledge, there is no effective biomarkers that can be used to predict the therapeutic effect of EOC and PSPC to bevacizumab treatment. Here we present a dataset of H&E WSIs with clinical information of EOC and PSPC patients, which will help researchers to explore and develop methods to predict the therapeutic effect of patients with EOC and PSPC to bevacizumab. This dataset is composed of de-identified 288 H&E stained WSIs (including 162 effective and 126 invalid WSIs) with clinical information of EOC and PSPC patients collected from 78 patients at the Tri-Service General Hospital and the National Defense Medical Center, Taipei, Taiwan. Examples of both effective and invalid treatment are shown in Fig. 1.

## Methods

### Study approval

The research ethics committee of the Tri-Service General Hospital approved the study (TSGHIRB No.1-107-05-171 and No.B202005070). All specimens were taken retrospectively from the histopathology archive of an author (TK, Chao) with approval by the Tri-Service General Hospital, National Defense Medical Center in Taipei, Taiwan (IRB approval ID No.1-107-05-171 and No.B202005070), and informed consent is formally waived by the approving committee.

### Instrumentation

The ovarian cancer WSIs were acquired with a digital scanner (Leica AT Turbo, Leica, Germany) with a 20× objective lens. The dimensions of WSIs collected in this work is 54342 × 41048 in pixels on average with the physical size 27.43 × 20.66 *mm*.

### Selection and preparation of specimen

**Fig. 2:**
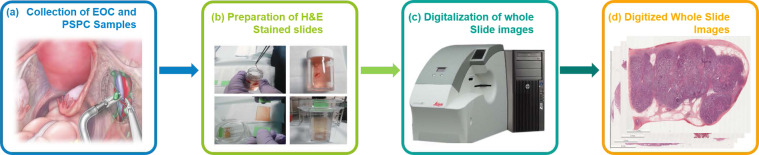
Generation of H&E stained WSIs. (**a**) EOC and PSPC samples from debulking surgery are collected. (**b**) H&E stained slides are prepared. (**c**) Stain slides are digitalized at the miscroscopic resolution. (**d**) Digitized WSIs are generated.

Specimens of EOC and PSPC had been surgically removed by the treating gynecologist for clinical suspicious of primary ovarian tumor. The tissue had been routinely fixed in formalin and embedded in paraffin. For this study tissue sections of treated effective and invalid groups were produced from the tissue blocks and staining with hematoxylin and eosin (Fig. 1) using an automated slide stainer (ST5010 Autostainer XL, Leica, Germany). Case selection was random and specimens with acceptable tissue quality were included. All images were digitized using a linear whole slide scanner (Leica AT Turbo, Leica, Germany) at a resolution of 0.5 microns per pixel (20X). Furthermore, all the images were examined by a pathologist who found them consistent and displaying no evidence of significant variations in intensity and color. Figure 2 shows the procedure followed for the generation of H&E stained WSIs.

## Data Records

### Data information

**Fig. 3:**
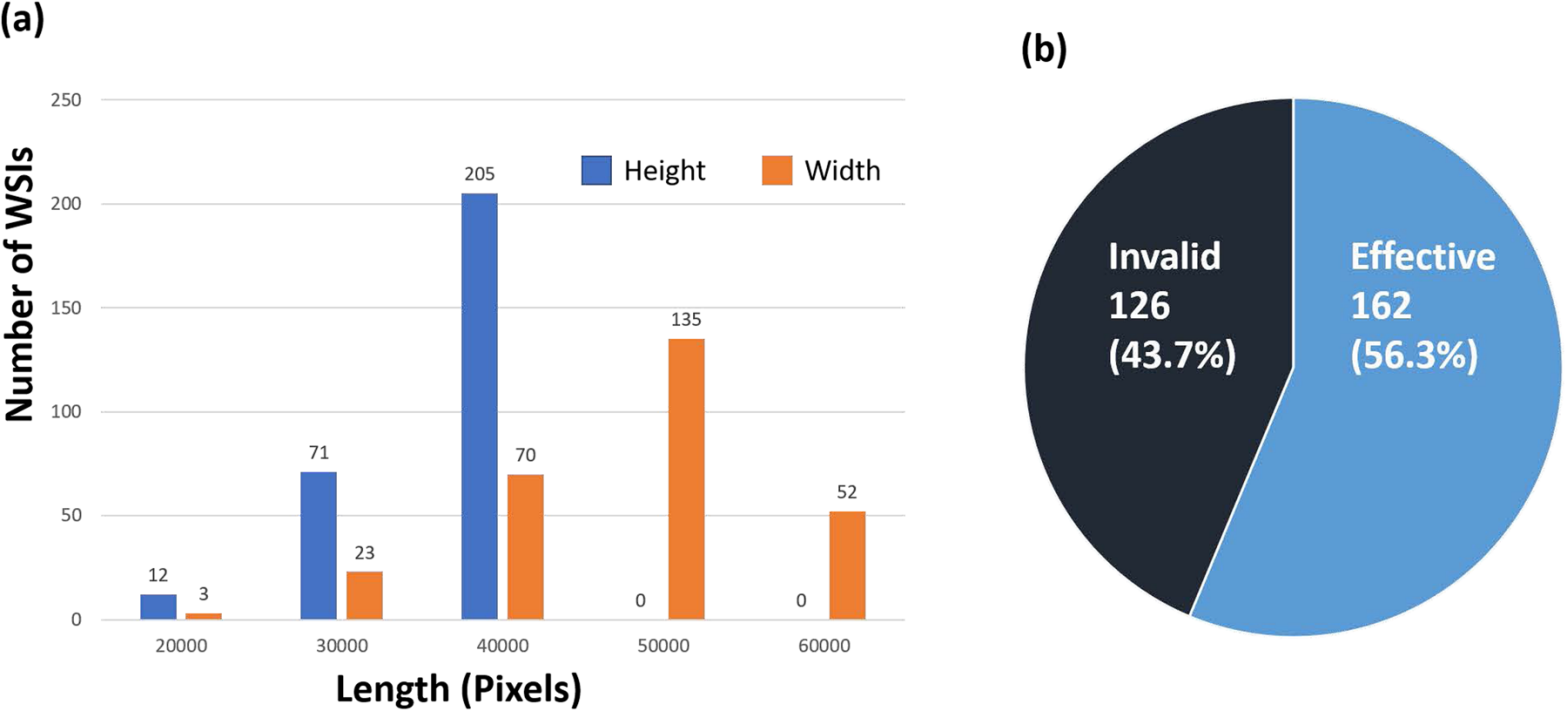
The dataset information. (**a**) Size distribution of slides heights(blue) and widths(orange). Distribution of effective and invalid WSIs.

Data has been accepted for publication on The Cancer Imaging Archive (TCIA). The dataset presented here is publicly available free-of-charge from the TCIA^17^. The dataset consists of 288 H&E stained WSIs, including 162 effective and 126 invalid WSIs were obtained from different tissue blocks of post-treatment specimens. Figure 3(a) shows the distribution of slide width and height in pixels.

### Patient list

The 288 slides are collected from 78 patients, and the list of patients along with the associated clinical information is provided in TCIA archive.

- **Patient ID** The unique patient ID of each patient.
- **Age** The age of each patient.
- **Diagnosis** The diagnostic result for each patient.
- **FIGO stage** The FIGO stage of each patient.
- **Operation** The process used to obtain specimen from each patient.
- **Method for avastin use** The method used for the administration of avastin.
- **Number of avastin administration** The number of avastin doses administered to each patient.
- **Operation date** The date on which specimen is collected from each patient.
- **Starting date for use of avastin** The date on which the first dose of avastin is administered to each patient.
- **End date for use of avastin** The date on which the last dose of avastin is administered to each patient.
- **Recurrent date** The recurrent date of each patient after bevacizumab therapy. If no recurrence of patient’s tumor, it is marked as “no recurrence”.
- **Date of death** The date of death of each patient. If patient survived the procedure, then survival is mentioned for that specific patient in the date of death column otherwise the date of death is mentioned in the column.
- **BMI** The body mass index of each patient.

### List of histopathological slides with CA-125 testing results

A list of 288 WSIs with associated patient ID, treatment effectiveness of bevacizumab therapy, and pre and post therapy CA-125 level is provided.

- **Patient ID** The unique patient ID of each patient.
- **Treatment effect** The effect of bevacizumab therapy to each patient.
- **Image No**. The number of each slide.
- **CA-125 before** The concentration of CA-125 U/ml (normal range from 0 to 35 U/ml) before clinical therapy.
- **CA-125 after** The concentration of CA-125 after clinical therapy.

## Technical Validation

To validate the proposed dataset, de-identified, digitized WSIs of 70 EOC and 8 PSPC patients (n = 78) including HGSOC (n = 58), endometrioid carcinoma (n = 4), clear cell carcinoma (n = 7), mucinous carcinoma (n = 2) and unclassied adenocarcinoma (n = 7) were obtained from the tissue bank of the Department of Pathology, Tri-Service General Hospital, National Defense Medical Center, Taipei, Taiwan. The clinicopathologic characteristics of patients were recorded by the data managers of the Gynecologic Oncology Center. Age, pre- and post-treatment serum CA-125 concentrations, histologic subtype, and recurrence status were recorded. These patients had received debulking surgery and chemotherapy with bevacizumab treatment. The regimen of chemotherapy with bevacizumab was based on the GOG-218, ICON-7, and GOG-213 trials. Patients with persistently high levels of CA-125 during bevacizumab therapy or who experienced tumor progression or recurrence (assessed by CT/PET imaging) within six months posttreatment were classified as the bevacizumab-resistant group. Patients with normal levels of CA-125 and no tumor progression or recurrence (based on imaging) during or within six months of bevacizumab treatment were classified as the bevacizumab sensitive group. Out of 288 patients slides, the bevacizumab treatment is effective for 162 slides (56.3%) and invalid for 126 slides (43.7%), as shown in Fig. 3(b).

## Data Availability

No code was used in the generation of this data. No code is required to access or analyze this dataset.

## References

[1] Siegel RL, Miller KD, Fuchs HE, Jemal A (2021). Cancer statistics, 2021. CA: a cancer journal for clinicians..

[2] Modugno, F. & Edwards, R. P. Ovarian cancer: prevention, detection, and treatment of the disease and its recurrence. molecular mechanisms and personalized medicine meeting report. *Int. J. Gynecol. Cancer* **22**. 10.1097/IGC.0b013e31826bd1f2 (2012). 10.1097/IGC.0b013e31826bd1f2 PMC3460381 23013733

[3] Torre LA (2018). Ovarian cancer statistics, 2018. CA: a cancer journal for clinicians.

[4] Prat J (2012). Ovarian carcinomas: five distinct diseases with different origins, genetic alterations, and clinicopathological features. Virchows Arch..

[5] Lheureux S, Braunstein M, Oza AM (2019). Epithelial ovarian cancer: evolution of management in the era of precision medicine. CA: a cancer journal for clinicians.

[6] Pentheroudakis G, Pavlidis N (2010). Serous papillary peritoneal carcinoma: unknown primary tumour, ovarian cancer counterpart or a distinct entity? a systematic review. Critical reviews oncology/hematology.

[7] Kurman RJ, Shih I-M (2011). Molecular pathogenesis and extraovarian origin of epithelial ovarian cancer—shifting the paradigm. Hum. pathology.

[8] Bignotti E (2007). Gene expression profile of ovarian serous papillary carcinomas: identification of metastasis-associated genes. Am. journal obstetrics gynecology.

[9] Kujawa KA, Lisowska KM (2015). Ovarian cancer–from biology to clinic. Postepy higieny i medycyny doswiadczalnej (Online).

[10] Coleman RL, Monk BJ, Sood AK, Herzog TJ (2013). Latest research and treatment of advanced-stage epithelial ovarian cancer. Nat. reviews Clin. oncology.

[11] Monk, B. J. & Coleman, R. L. Changing the paradigm in the treatment of platinum-sensitive recurrent ovarian cancer:from platinum doublets to nonplatinum doublets and adding antiangiogenesis compounds. *Int. J. Gynecol. Cancer* **19**. 10.1111/IGC.0b013e3181c104fa (2009). 10.1111/IGC.0b013e3181c104fa 19955917

[12] Cortez AJ, Tudrej P, Kujawa KA, Lisowska KM (2018). Advances in ovarian cancer therapy. Cancer chemotherapy pharmacology.

[13] Lalwani N (2011). Histologic, molecular, and cytogenetic features of ovarian cancers: implications for diagnosis and treatment. Radiogr..

[14] Al-Janabi S, Huisman A, Van Diest PJ (2012). Digital pathology: current status and future perspectives. Histopathol..

[15] Stathonikos, N., Veta, M., Huisman, A. & van Diest, P. J. Going fully digital: Perspective of a dutch academic pathology lab. *J. pathology informatics* **4**. https://www.jpathinformatics.org/text.asp?2013/4/1/15/114206 (2013). 10.4103/2153-3539.114206 PMC3709427 23858390

[16] Bankhead P (2017). Qupath: Open source software for digital pathology image analysis. Sci. reports.

[17] Wang C-W (2021). The Cancer Imaging Archive..

